# Heat-Moisture Treatment Further Reduces In Vitro Digestibility and Enhances Resistant Starch Content of a High-Resistant Starch and Low-Glutelin Rice

**DOI:** 10.3390/foods10112562

**Published:** 2021-10-24

**Authors:** Zhiyuan Li, Dongshu Guo, Xiao Li, Zhaocheng Tang, Xitie Ling, Tiantian Zhou, Baolong Zhang

**Affiliations:** 1 School of Food and Biological Engineering, Jiangsu University, Zhenjiang 212013, China; 2221918037@stmail.ujs.edu.cn; 2 Excellence and Innovation Center, Jiangsu Academy of Agricultural Sciences, Nanjing 210014, China; guods_cau@163.com (D.G.); lixiao_jaas@163.com (X.L.); 18816201386@163.com (Z.T.); xtling0129@163.com (X.L.); 2021116027@stu.njau.edu.cn (T.Z.)

**Keywords:** *Oryza sativa* L., rice flour, heat-moisture treatment, in vitro digestibility, resistant starch

## Abstract

A novel rice germplasm *sbeIIb*/*Lgc1* producing grains rich in resistant starch (RS) but low in glutelin has been developed through CRISPR/Cas9-mediated targeted mutagenesis for its potential benefits to patients with diabetes and kidney diseases. In this study, a hydrothermal approach known as heat-moisture treatment (HMT) was identified as a simple and effective method in reinforcing the nutritional benefits of *sbeIIb*/*Lgc1* rice. As a result of HMT treatment at 120 °C for 2 h, significant reductions in in vitro digestibility and enhancements in RS content were observed in *sbeIIb*/*Lgc1* rice flour when the rice flour mass fraction was 80% and 90%. The low-glutelin feature of *sbeIIb*/*Lgc1* rice was not compromised by HMT. The potential impacts of HMT on a range of physicochemical properties of *sbeIIb*/*Lgc1* rice flour have also been analyzed. HMT resulted in a darker color of rice flour, alteration in the semi-crystalline structure, an increase in gelatinization temperatures, and reductions in the pasting viscosities as the moisture content increased. This study provides vital data for the food industry to facilitate the application of this dual-functional rice flour as a health food ingredient.

## 1. Introduction

Rice is one of the most important cereal crops worldwide and the processed rice grain, known as white rice, contains mainly starch as its nutritional component [1,2,3]. Starch is a polymeric carbohydrate consisting of multiple glucose units that are joined by glycosidic bonds [4,5]. Starch consists of two distinct types of polymers, i.e., the almost linear amylose and highly branched amylopectin, based on the glycosidic bond type and branch formation [4,5]. Owing to the different intrinsic features of amylose and amylopectin at the molecular level and consequent different reactions to varied processed methods, the ratio of amylose/amylopectin determines the physicochemical properties and nutritional values of the processed starchy material [6,7]. The natural starches of cereal grains, tubers and legumes exist in a semi-crystalline form including the crystalline region and amorphous region [4,5]. Generally, the starch crystalline structures were classified into A-type, B-type and the C-type, which is considered a mixture of A- and B-types [8,9]. It has been reported that B-type starch is less prone to digestion because of its structural properties [10]. However, the white rice produced by most rice cultivars contains predominately A-type starch [9,11]. The concept of resistant starch (RS) was first proposed four decades ago based on its digestibility [12]. RS is defined as a form of starch that is resistant to enzymatic hydrolysis and barely digested in human small intestine; instead, it is fermented in the large bowel by colonic bacteria to produce short-chain fatty acids (SCFAs) that have been proposed to have potential benefits for the health of intestinal tract [10,11]. Owing to the high level of resistance to digestion, a diet rich in RS helps to reduce postprandial blood glucose levels and alleviate insulin resistance for patients with diabetes [10,11]. Because most white rice contains only trace amounts of RS [3,13,14], it is desirable to enhance its RS content which is imperative for preventing diabetes and improving the health status of diabetes sufferers [11,13,15,16].

Protein is second only to starch as an important nutritional component in a rice grain [3,17], 90% of which are storage proteins [17,18]. Glutelin (60~80%) and prolamin (10~20%) are the most abundant among the rice storage proteins [3,19]. Glutelin and prolamin are located in distinctive protein bodies, and the former is relatively easier to digest than the latter in the human digestive system [18]. Considering the digestion properties of different protein fractions, rice grains with a lower glutelin content are considered desirable to alleviate the metabolic burden of patients with kidney diseases [20,21,22].

In previous studies, our group and others have generated rice lines with higher than normal RS contents in both *japonica* and *indica* by disrupting the expression of the rice *starch branching enzyme IIb* (*SBEIIb*) gene that controls the synthesis of amylopectin through chemical mutagenesis [23,24], RNA interference [25,26] and genome editing [13,14]. In particular, we have mutated the *SBEIIb* gene in the background of an elite low-glutelin rice cultivar derived from LGC-1 [21,27], leading to the development of *sbeIIb*/*Lgc1* that harbors both high-RS and low-glutelin features [13]. The *sbeIIb* rice lines were found to have B-type starch grains and the apparent amylose content (AAC) of them was close to or above 30%, which is significantly higher relative to the corresponding wild type parent line [13,14,23,24,25,26]. It has also been demonstrated that the RS content of the freshly cooked rice or native flour derived from *sbeIIb* rice was significantly higher than that of common rice [13,14,25]. Thus, it has been widely considered that *sbeIIb* rice is a promising food stock for patients with diabetes [13,14,25,26]. However, the texture of the freshly steamed *sbeIIb* rice was rather hard, rendering it barely palatable [13]. To our knowledge, so far there has been no research on the comprehensive analyses of the digestibility of *sbeIIb* rice flour and exploration of *sbeIIb* rice as an ingredient in processed food. Further, the genetic improvement on the high RS content and low hydrolysis rate in *sbeIIb*/*Lgc1* and other *sbeIIb* rice germplasms of different genetic backgrounds may not always reach the desired levels to meet the specific needs of diabetes patients [13,14,25]. Taken together, these warrant exploration of food processing methods for further reinforcement in the slow digestion feature of such high-RS germplasms.

Both chemical and physical methods have been reported to be able to modify the physicochemical properties of cereal starch [28,29]. Compared with chemical modifications that need additional chemical reagents, hydrothermal treatment as a widely used physical modification method is considered to be more readily accessible and environmentally friendly [28,29]. Among different hydrothermal treatment methods, heat-moisture treatment (HMT), which applies low moisture content (generally below 35%) and high temperature (generally 80~140 °C) for certain durations, has been proven to be effective in changing the susceptibility of cereal starch to α-amylase [29,30]. HMT affects the crystalline structure of starch and changes the interactions of starch chains in the amorphous and crystalline regions [30]. In many cases, the proportion of slowly digestible starch (SDS) and RS content increased to various extents following HMT, but in other cases, HMT raised the digestibility of starch [29,30,31]. The incongruence and variations in the effects of HMT on starch digestibility in the literature could well be attributed to a number of factors, such as different plant species or genetic background, the innate properties of starch (e.g., amylose content, starch crystalline structure, starch granule size), the content of other components (e.g., protein and lipids), heat sources, and the parameters of HMT [29,32]. In rice, the overwhelming majority of studies have focused on the conventional cultivars with typical A-type crystalline structure and low RS content [6,32,33]. There is an apparent lack of information on the effects of HMT on the in vitro digestibility and the physicochemical properties of the recently developed *sbeIIb* rice flour with B-type starch crystalline structure and high RS content.

For the reason of direct use as a food ingredient, and the consideration of the possible presence of type 5 RS due to the high lipid content of *sbeIIb*/*Lgc1* rice [10,13], we conducted HMT experiments by using rice flour instead of purified starch to evaluate the potential effects of HMT on in vitro digestibility and RS content of *sbeIIb*/*Lgc1* rice. Further, the underlying mechanisms by which HMT affects the physicochemical properties and digestibility of *sbeIIb*/*Lgc1* rice flour were investigated by analyzing the color and chromaticity, the morphology of starch granules, the crystalline structures, the gelatinization properties and the pasting properties of HMT treated flours to provide comprehensive information for the food manufacturers to rationally and flexibly employ this dual functional rice flour into versatile health food products.

## 2. Materials and Methods

### 2.1. Materials

The *sbeIIb*/*Lgc1* rice with high-RS and low-glutelin features was generated by disrupting the *SBEIIb* gene (*LOC_Os02g32660*) in a rice cultivar derived from a low-glutelin germplasm Low Glutelin Content-1 (LGC-1) [21,27] using a CRISPR/Cas9 system [13]. Suxianggeng 100 (SXG100), an elite *japonica* rice cultivar with normal RS and gluten contents, was used as a common rice control. The common nutritional components of SXG100 include total starch (~83.08%), AAC (~10.18%), RS (~0.06%) and crude lipids (~0.46%). The homozygotes of *sbeIIb*/*Lgc1*-6-6, *sbeIIb*/*Lgc1*-28-3 [13], and SXG100 were planted in the field during the growing season in 2019 in Nanjing, Jiangsu, China.

### 2.2. Rice Flour Preparation

Newly harvested rice grains were air dried for about a month before being polished and milled. Passing through a 100-mesh sieve, the rice flour was equilibrated at 45 °C for about 24 h to reach the moisture of ~12% before heat treatment.

### 2.3. Heat Treatment

Distilled water was added to the freshly prepared rice flour in silk mouth bottles with a gradient of flour mass fractions of 100% (40 g rice flour), 90% (36 g rice flour/4 g water), 80% (32 g rice flour/8 g water) and 70% (28 g rice flour/12 g water). Sealed with parafilm (PM-996, Bemis, Shirley, MA, USA), the bottles were stored at room temperature for 24 h for moisture equilibration. Following heating in an oil bath at 120 °C for 2 h, the samples were cooled down to room temperature before being dried at 45 °C for 24 h. The dried samples were ground and sieved through a 100-mesh sieve to prepare samples for further analyses. The accurate moisture content was then determined by a HB43-S Moisture Analyzer (Mettler-Toledo AG Laboratory & Weighing Technologies, Greifensee, Switzerland). Rice flour that was not subjected to heat treatment was considered as the native rice flour. It is worth noting that a rice flour mass fraction of 70% in which the moisture content was slightly higher than the conventional standard of HMT was set to facilitate the mechanism analysis of changes caused by HMT, and, for brevity, the heat-treatment at flour mass fraction of 70% is also referred as HMT in this article.

### 2.4. In Vitro Hydrolysis Experiments

The digestibility of native or HMT flour was evaluated by measuring the released glucose as described previously [34] with some modifications. Briefly, to prepare the enzyme solution, 2.5 g porcine pancreas α-amylase (A3176, Sigma-Aldrich, St Louis, MO, USA) and 100 mg glucosidase (T8500, Solarbio Life Sciences, Beijing, China) were added into 100 mL deionized water and mixed by magnetic stirring for 10 min, prior to centrifugation at 1500× *g* for 10 min, and the supernatant was collected and used as the enzyme solution. In total, 5 mL of the above-prepared enzyme solution and 7.5 mL of 0.5 M sodium acetate buffer (pH 5.2) were added to a 50 mL screw-capped tube containing 100 mg flour and mixed thoroughly. Then, the samples were incubated at 37 °C by shaking at 220 rpm. The aliquots of 200 μL were taken at 60, 120 and 180 min of incubation and immediately mixed with 800 μL absolute ethanol to terminate the reaction. After the centrifugation at 1500× *g* for 10 min, the supernatant was collected to quantify the released glucose by the D-Glucose assay kit (K-GLUC, Megazyme, Ireland) following the manufacturer’s instructions. The absorbance at 510 nm was measured by using a Spark^®^ microplate reader (Tecan Group Ltd., Männedorf, Switzerland). The amount of the released glucose in test samples measured as mg/100 mg flour was calculated as follows: Released Glucose = [Absorbance (sample) − Absorbance (assay blank)]/[Absorbance (1 mg/mL D-glucose solution) − Absorbance (reagent blank)] × 5 × 12.5.

### 2.5. RS Content Determination

The RS contents of native and HMT flours were measured using a resistant starch assay kit (K-RSTAR, Megazyme, Ireland), referring to the Association of Official Analytical Chemists (AOAC) official method 2002.02. To determine the RS content of gelatinized flour, a sample of 100 mg native or HMT flour was mixed with 1 mL distilled water and then boiled at 100 °C for 20 min before measurement using the K-RSTAR kit. When the gelatinized samples were cooled down to room temperature, the sample was used for the measurement using the K-RSTAR kit immediately.

### 2.6. Color and Chromaticity Analyses

A 24-well plate containing individual samples of native or HMT flour of 500 mg in weight was photographed using a Nikon D90 digital single-lens reflex (DSLR) camera (Nikon Corporation, Tokyo, Japan). The chromaticity of each sample was determined by using a spectrocolorimeter CM-700d (Konica Minolta, Inc., Tokyo, Japan) following the manufacturer’s instructions. The total color difference (ΔE) and whiteness index (WI) were calculated as following formulas [35]:

$$\Delta E=\sqrt{\Delta L^{*2}+\Delta a^{*2}+\Delta b^{*2}}$$

$$\mathrm{WI}=100\;-\;\sqrt{{\left(100\;-\;L^{*}\right)}^{2}+a^{*2}+b^{*2}}$$

### 2.7. Starch Extraction

Approximately 1 g native or HMT flour was added into 4 mL of a 4 g/L NaOH solution prior to being stirred for 2 h. After centrifugation at 3000× *g* for 15 min, the yellow supernatant was removed. The pellet was washed with distilled water three times by centrifugation at 3000× *g* for 15 min. Following the removal of the supernatant, the pellet was dried at 55 °C for 24 h to obtain coarse starch. The dried coarse starch was then dissolved into 4 mL of a 5 g/L alkaline protease solution (B8360, Solarbio, China), the pH value of which was adjusted to 10 with 1 M NaOH, prior to being stirred at 45 °C for 2 h. After centrifugation at 3000× *g* for 15 min and removal of the supernatant, the pellet was washed with distilled water three times by centrifugation at 3000× *g* for 15 min. Following the removal of the supernatant, the pellet was washed with 1 mL methanol three times by free sedimentation for 1 h and centrifuged at 10,000× *g* for 10 min. Following the removal of the supernatant after the last wash, the pellet was dried at 55 °C for 24 h prior to grounding and sieving through a 100-mesh sieve to obtain pure starch.

### 2.8. Scanning Electron Microscopy

The pure starch powder was mounted to an aluminum stub using double-sided adhesive tape and coated with gold prior to morphological observation of starch granules by using a scanning electron microscope (SEM) Zeiss EVO-LS10 (Carl Zeiss, Jena, Germany) following the manufacturer’s instructions.

### 2.9. X-ray Diffraction (XRD)

The native and HMT flours were filtered by a 100-mesh sieve prior to being exposed to the monochromatic Cu-Kα radiation (λ = 0.15406 nm) produced by an X-ray powder diffractometer Bruker D2 PHASER (Bruker Labscape, Ettlingen, Germany) at 40 kV and 40 mA. The scanning regions of the diffraction angle 2θ were 5–60°. The scan rate was 4°/min with a step interval of 0.02°. The relative crystallinity was calculated and the original XRD patterns were processed by the software EVA 4.1 (Bruker Labscape, Ettlingen, Germany) referring to the method described previously [36]. The resolution of multiple diffraction peaks was conducted using Jade 6.2 referring to the method described previously [37].

### 2.10. Gelatinization Properties Analyses

Following filtration by a 100-mesh sieve, a sample of 100 mg native or HMT flour was equilibrated and dried at 37 °C for 24 h, 5 mg of which was mixed with 10 μL deionized water in a hermetical aluminum cup prior to being heated from 20 °C to 110 °C at a rate of 10 °C/min for the native and HMT rice flours of SXG100, and from 20 °C to 130 °C at a rate of 10 °C/min for the native and HMT rice flours of *sbeIIb*/*Lgc1*. The gelatinization properties were analyzed using a Differential Scanning Calorimeter (DSC) DSC-Q20 (TA Instruments, New Castle, DE, USA).

### 2.11. Pasting Properties Analyses

Distilled water was added to a series of samples of native and HMT flours which were filtered by a 100-mesh sieve, and then measured by a rapid visco analyzer RVA4800 (Perten Instruments, Stockholm, Sweden). The standard moisture content was set as 14%, under which the amounts of flour and water added were 3 g and 25 g, respectively. For each native and HMT sample, the amounts of flour and water were calculated by the built-in calculator of the RVA4800 software (TCW 3.17.4.514, Perten, Australia), depending on the actual moisture content. The rotation speed of the paddle was set to 160 rpm, and the resulting flour slurries were then maintained at 50 °C for 1 min before being heated from 50 °C to 95 °C at a rate of ~12.2 °C/min, at which they were maintained for 3.5 min before cooling down to 50 °C at a rate of ~12.2 °C/min, and finally maintained at 50 °C for another 2 min.

### 2.12. Protein Extraction and SDS-PAGE

A sample of 100 mg native or HMT flour was dispersed in 800 μL extraction solution (4% *w*/*v* SDS, 4 M Urea, 5% *v*/*v* β-mercaptoethanol, 125 mM Tris-HCl) [38], and incubated for 30 min at room temperature with gentle shaking. After centrifugation at 13,000 rpm for 15 min at 4 °C, the supernatant was transferred to a new tube and mixed with loading buffer prior to being heated at 100 °C for 5 min to denature the protein, 15 μL of which was fractionated by SDS-PAGE (5% stacking gel/12% separation gel). The gel with fractionated proteins was stained by coomassie brilliant blue solution (0.1% *w*/*v* coomassie brilliant blue R-250, 25% *v*/*v* isopropanol, 10% *v*/*v* glacial acetic acid) for about 3 h at room temperature. Following the treatment by a destaining solution (10% *v*/*v* glacial acetic acid, 5% *v/v* ethanol) overnight, the protein gel was photographed by using a Nikon D90 DSLR camera (Nikon Corporation, Tokyo, Japan).

### 2.13. Preparation of Steamed Rice

A sample of 10 g of the polished rice grains of *sbeIIb*/*Lgc1* or SXG100 was soaked in 20 mL tap water for 30 min in a glass beaker and sealed by a piece of filter paper prior to being steamed on boiling water for 30 min. The freshly steamed rice was photographed by a Nikon D90 DSLR camera (Nikon Corporation, Tokyo, Japan).

### 2.14. Statistical Analyses

All experiments were conducted with three replications, except the analysis of chromaticity which was conducted with nine replications. Statistical analyses were conducted using IBM Statistics 22.0 (IBM Inc., Armonk, NY, USA). The significance of variation in RS content of gelatinized or ungelatinized flours was analyzed using F-test to detect the homogeneity of variances, followed by a two-tailed t test, while the other measured values were analyzed by ANOVA and compared by least significant difference (LSD) analysis. *p* < 0.01 was considered as being significantly different for all the comparisons.

## 3. Results and Discussion

### 3.1. In Vitro Digestibility

Following treatment by α-amylase and amyloglucosidase in combination, the amount of released glucose at different time points (60, 120 and 180 min) of digestion in the native or HMT rice flour was measured to predict the changes of digestibility (Table 1). It is apparent that the amount of the released glucose from native *sbeIIb*/*Lgc1* rice flour was significantly lower than that of SXG100 at all the time points tested. This result is consistent with previous research about *sbeIIb* [14]. For the HMT rice flours of *sbeIIb*/*Lgc1*, the amount of released glucose at a rice flour mass fraction of 100% was comparable to that of *sbeIIb*/*Lgc1* native flour. When the rice flour mass fraction was 90% and 80%, the glucose release amounts of HMT *sbeIIb*/*Lgc1* rice flour decreased gradually, and at the time point of 120 min, the amounts of released glucose were ~72% and ~64% of that of native *sbeIIb*/*Lgc1* rice flour, respectively. When the rice flour mass fraction of *sbeIIb*/*Lgc1* was 80%, the starch hydrolysis rate at 120 min of digestion was only about 30%, which was the lowest among all the tested samples. According to the above results, we demonstrated that when the rice flour mass fraction was 90% and 80%, HMT at 120 °C for 2 h could further lower the in vitro digestibility of *sbeIIb*/*Lgc1* rice flour, and they were expected to have better performance than native rice flour when used as an ingredient of food for diabetic patients.

When the rice flour mass fraction of *sbeIIb*/*Lgc1* was reduced down to 70% under which the moisture content was slightly higher than conventional HMT standard, the amount of released glucose was higher than that of native flour. Such an observation might be attributed to the severe impact on the starch granule structure by high intensity heat treatment and the occurrence of partial gelatinization [7,33,39], even though the amount of released glucose of HMT *sbeIIb*/*Lgc1* at a rice flour mass fraction of 70% was still lower than that of native rice flour of SXG100. Considering the energy supply, the lowest digestibility may not be always desirable. Through in vitro digestion assay, this study established that the effects of HMT on digestibility vary by the moisture content of treated rice flour. Therefore, the moisture content of HMT flour could be adjusted to achieve the optimal digestion rate to suit specific dietary requirements.

The effect of HMT on the digestibility of SXG100 rice flour was also observed, which showed a similar trend of reduction in hydrolysis rate, but to a lesser extent compared to *sbeIIb*/*Lgc1*. Under HMT of 100% rice flour mass fraction, the digestion rate of the SXG100 flour began to decrease, and the lowest amount of released glucose for SXG100 was observed when the rice flour mass fraction was 90%. In contrast, when the rice flour mass fraction was 100%, the digestibility of HMT *sbeIIb*/*Lgc1* rice flour was comparable with that of native flour and decreased when the moisture content increased. This might well be attributed to the characteristics of *sbeIIb*/*Lgc1* starch granules and starch molecules which require more water to alter the interactions of starch molecules [29]. Such a hypothesis is supported by SEM observations that when the rice flour mass fraction was 80%, the morphology of the starch granules from HMT *sbeIIb*/*Lgc1* rice flour was still recognizable but those of SXG100 had already begun to disintegrate and fuse together (Figure 3).

### 3.2. Resistant Starch Content

**Figure 1:**
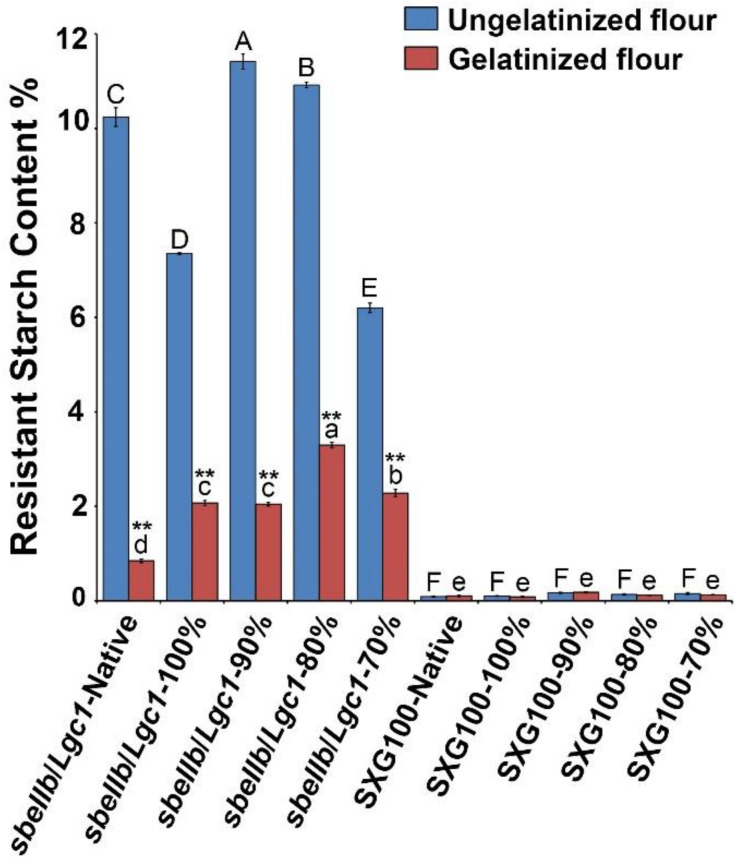
Resistant starch contents of native and HMT rice flours of *sbeIIb*/*Lgc1* and SXG100. All the analyses were repeated three times. Data are presented as mean ± standard derivation. ANOVA and the least significant difference method were used to conduct statistical analyses of gelatinized or ungelatinized flours. Different uppercase letters represent the significant difference of RS content of ungelatinized flours, while different lowercase letters represent those of gelatinized flours. *p* < 0.01 was considered as being significantly different. A two-tailed *t*-test was used to conduct statistical analyses of gelatinized and ungelatinized treatments under the same condition. ** represents *p* < 0.01.

In addition to in vitro digestibility, the comparative analysis of the RS contents of native and HMT flours is shown in Figure 1. For the native flours, the RS content of *sbeIIb*/*Lgc1* rice flour was about 10.24% which was in sharp contrast to that of SXG100 (0.09%) which is in line with a previous study [13]. For HMT rice flour of *sbeIIb*/*Lgc1*, the RS content of the ungelatinized rice flour of 100% mass fraction was significantly lower than that of the native flour, which might be partially attributed to the disruption in the crystalline structure as reflected by the decrease of relative crystallinity (Figure 4). As the rice flour mass fraction decreased, the RS content was significantly raised relative to native flour at both rice flour mass fractions of 90% and 80% as a result of heat treatment. It might be that as the moisture content of treated rice flour increased, more water molecules made entry into the starch granules, which might alter the interactions of starch molecules to a great extent and reinforce the entanglements between starch chains or starch with other molecules, e.g., lipid or protein [29,33]. At the rice flour mass fraction of 70%, the RS content was decreased to the lowest level in the tested *sbeIIb*/*Lgc1* samples, which might also be caused by the partial gelatinization. The above observations demonstrated that HMT played a substantial role in the further increase of RS content of *sbeIIb*/*Lgc1* when the moisture content of the sample was selected appropriately.

The RS contents of the gelatinized native and HMT rice flours were also determined to facilitate the investigation of the mechanisms by which RS formed. Following gelatinization at 100 °C for 20 min, the RS contents of both the native and HMT rice flours of *sbeIIb*/*Lgc1* were drastically reduced relative to their ungelatinized counterparts. Since gelatinization will cause an irreversible collapse of the starch granules and crystalline structures [31], this observation suggests that the high RS content of ungelatinized native and HMT *sbeIIb*/*Lgc1* rice flour might be mainly attributed to the crystalline structure of starch and the products formed during HMT with resistance to amylase but lower thermal stability [10,29]. Nevertheless, the RS contents of gelatinized HMT *sbeIIb*/*Lgc1* rice flours were significantly higher than that of native flour with the highest content being 3.30% at the flour mass fraction of 80%. For SXG100, though a slight enhancement of RS content was also observed when the sample moisture content was higher, the enhancement was not statistically significant and the absolute RS amounts were still quite low compared with those of *sbeIIb*/*Lgc1*. These observations revealed that HMT had a greater effect on *sbeIIb*/*Lgc1* than SXG100 on reinforcing the interactions between starch molecules and promoting the formation of the thermally stable amylose-lipid complex that contributes to RS5, which might be attributed to higher AAC content and lipid content in *sbeIIb*/*Lgc1* compared to SXG100 [6,7,40].

It has been previously reported that the RS content of the freshly cooked *sbeIIb* rice was about 5~9%, varying among independent lines [14], which is higher than those of the gelatinized native flour in our research. Such a discrepancy might be caused by the variation in the food processing methods which were used in these two independent experiments. This is not unreasonable as the hard texture of the whole grain of *sbeIIb* rice makes it quite difficult to cook. In contrast to SXG100, the rice grains of *sbeIIb*/*Lgc1* largely maintained their original morphology following the cooking process, with the core of many grains remaining undercooked even after being steamed at 100 °C for 30 min (Figure S1). Thus, the measured RS content of the freshly cooked *sbeIIb* rice would be higher than that in the gelatinized native *sbeIIb*/*Lgc1* rice flour.

### 3.3. Color and Chromaticity

Both the direct visualization and chromaticity quantification with a colorimeter were performed to evaluate the color changes of the rice flour after HMT. L*-, a*-, b*-values were used to quantitatively describe the color of rice flour. L*-value indicates darkness to lightness; a*-value indicates greenness to redness; and b*-value indicates blueness to yellowness. The total color difference (ΔE) was calculated to describe the extent of color difference of two samples and the whiteness index (WI) was calculated to describe the closeness of a sample to white [35]. It has been reported that untrained people can distinguish slight color difference indicated by the ΔE > 2 and clear difference indicated by the ΔE > 3.5 [35].

**Figure 2:**
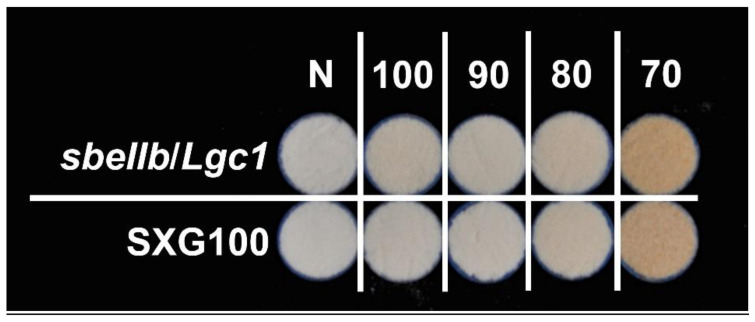
The appearances of native and HMT flours of *sbeIIb*/*Lgc1* and SXG100 rice. N represents native flour. The numbers on the upper part of the figure represent the rice flour mass fraction (%) of samples subjected to HMT.

The appearances of native and HMT rice flours are shown in Figure 2 and the results of chromaticity analyses are listed in Table 2. Accordingly, the native rice flour of *sbeIIb*/*Lgc1* was slightly darker and browner than that of SXG100, possibly because of the different chemical compositions. As a result of HMT, the color of HMT rice flours turned darker progressively as reflected by the L*-value for both *sbeIIb*/*Lgc1* and SXG100. Meanwhile, the increases in the a*-value and b*-value indicated that the degrees of redness and yellowness of the HMT flours of both *sbeIIb*/*Lgc1* and SXG100 were also enhanced. Consequently, the WI of HMT rice flours was also gradually decreased as the moisture content increased. A similar observation was also made when rice flour was treated by HMT under some other conditions [41,42]. According to Figure 2 and ΔE in Table 2, the color changes of HMT flours were not so great when the rice flour mass fractions were 100~80% as those when the rice flour mass fractions were 70%. It has been proposed that the changes in flour color might be attributed to the amino-carbonyl reactions that occurred between proteins and amino acids with carbonyl-containing compounds during heat treatment [41,42]. Under the same HMT condition, the HMT rice flours of *sbeIIb*/*Lgc1* were always brown and darker than those of SXG100, but for specific treatment the color difference between *sbeIIb*/*Lgc1* and SXG100 was not dramatically higher than that between two native flours as reflected by comparable ΔE values when the rice flour mass fraction was 100% and 90%, suggesting the similar extent of effects of HMT on these two rices in spite of their difference in chemical composition. It has been reported that the extent of the browning of rice flour was positively correlated to the intensity of HMT [41,42]. Therefore, the parameters of HMT could be adjusted to suit the specific requirements of food coloration.

Interestingly, when the rice flour mass fraction was 80%, the ΔE between the HMT samples of *sbeIIb*/*Lgc1* and SXG100 was only 0.8 and the ΔE between HMT and native flours of SXG100 was higher than that of *sbeIIb*/*Lgc1*. Considering the great color changes of HMT rice flours when the mass fraction was 70%, this observation suggested a promoting effect of partial gelatinization on the browning of heat-treated rice flours.

### 3.4. Starch Granule Morphology

**Figure 3:**
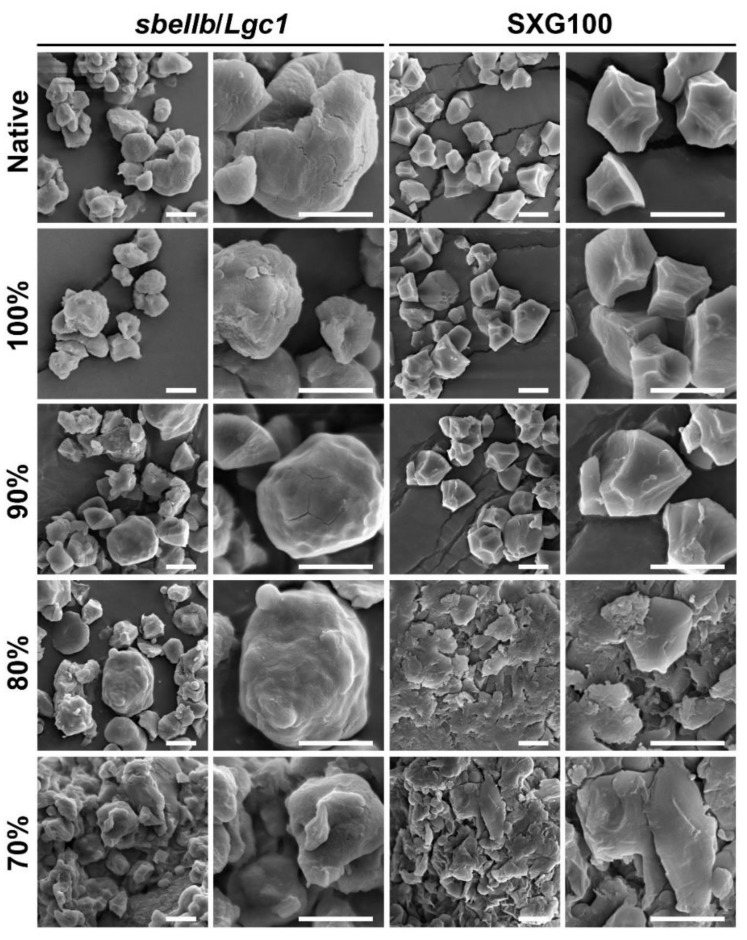
Morphology of starch granules of native and HMT flours observed by the SEM. Bars = 5 μm. For each sample, the images of representative starch granules with the magnification of 2000× and 5000× were presented.

To determine the effects of HMT on the morphology of starch granules, the starch derived from native and HMT flours was observed by SEM as shown in Figure 3. Distinct from the regular polyhedral starch granules of SXG100, which is typical in common rice, the starch granules of *sbeIIb*/*Lgc1* showed irregular shapes with variable sizes, which is consistent with a previous study [13].

After HMT, neither the morphology nor the integrity of starch granules of *sbeIIb*/*Lgc1* was severely impacted as the intact starch granules could be recognized readily when the rice flour mass fraction was above 80%. As the moisture further increased, i.e., when the rice flour mass fraction was 70%, the integrity of starch granules of *sbeIIb*/*Lgc1* was impaired to a large extent and the individual starch granules began to agglomerate and fuse into clumps. A similar tendency was observed for the starch of HMT flours of SXG100. However, when the rice flour mass fraction was decreased to 80%, the starch granules in SXG100 HMT flour already showed severe collapse and fusion. These observations demonstrated that when the moisture content reached a threshold level, the physical integrity of starch granules collapsed to a great extent as the result of partial gelatinization, which is in agreement with previous reports [6,33,39].

It is worth noting that when the flour mass fraction was 80%, which is considered as HMT, the starch granules of SXG100 had already collapsed and fused together, while the integrity of the starch granules from *sbeIIb*/*Lgc1* was maintained to a large degree. Taken together, these observations suggested that the starch granules of *sbeIIb*/*Lgc1* were more resistant to gelatinization inflicted by HMT with higher moisture content, which might be attributed to the high ACC of *sbeIIb*/*Lgc1* [13]. This observation might also account for the observed difference in in vitro digestibility between *sbeIIb*/*Lgc1* and SXG100 HMT flours when the flour mass fraction was 80% and 70%.

### 3.5. Crystalline Structure

**Figure 4:**
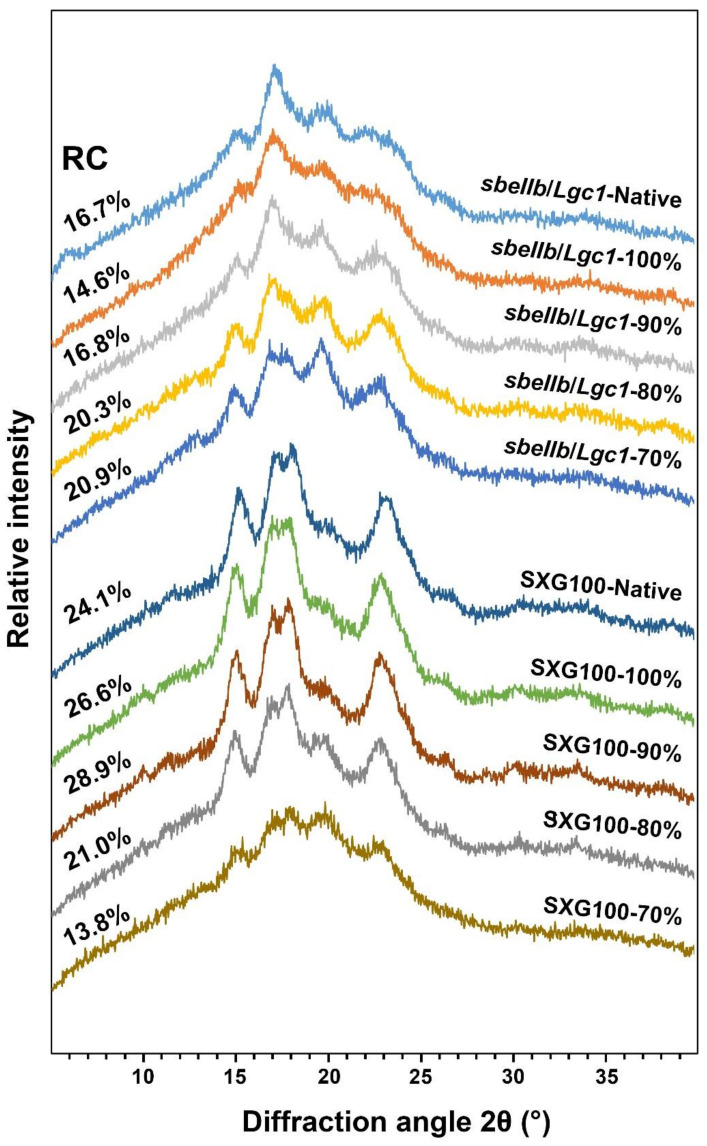
X-ray diffraction patterns of the native and HMT rice flours of *sbeIIb*/*Lgc1* and SXG100. The percentages of the left part of the penal indicate the relative crystallinity (RC).

XRD assay was conducted to analyze the changes in the crystalline type and relative crystallinity of rice flours after HMT. As shown in Figure 4, Figures S2 and S3, the diffraction pattern of native *sbeIIb*/*Lgc1* flour resembled B-type as manifested by the diffraction peaks at 2θ angles of 15°, 17°, 22°, 24° and ~5.6°, whereas the diffraction pattern of native SXG100 flour was a typical A-type manifested by the diffraction peaks at 2θ angles of 15°, 17°, 18°, and 23°. It appears that the XRD patterns of the native rice flour of both *sbeIIb*/*Lgc1* and SXG100 were comparable to those of pure starches [13]. For *sbeIIb*/*Lgc1*, when the rice flour mass fraction was 100%, the relative crystallinity decreased and the feature peak of B-type crystalline at ∼5.6° 2θ angle disappeared. Intriguingly, the diffraction peak at the 17° 2θ angle appeared to split while the shoulder peaks at 22° and 24° showed signs of fusion when the rice flour mass fraction was 90%, and the B-type polymorph crystallites in native *sbeIIb*/*Lgc1* rice flour transitioned to A-type when the rice flour mass fraction was reduced to 70%, as manifested by the diffraction peaks at 15°, 17°, 18° and 23° 2θ angles (Figure 4 and Figure S2). Concomitantly, the relative crystallinity of HMT *sbeIIb*/*Lgc1* rice flours gradually increased when the rice flour mass fraction was 90% to 70%. Similar observations have also been made in other B-type starches of tuber and legume plants under thermal treatments [30,43,44]. The alteration in the crystalline structure of starch might be attributed to the rearrangement of starch chains during the process of HMT [30,43]. Notably, the intensity of a diffraction peak at 2θ angle of ~20° became gradually stronger in the course of HMT treatment for *sbeIIb*/*Lgc1*, suggesting the increasing presence of the starch–lipid complex [29,36], which might be positively related to the increased RS content in the gelatinized HMT rice flour (Figure 1).

In contrast, for SXG100, the A-type crystalline structure was barely affected by HMT when the flour mass fraction was 100% to 80%, which is in agreement with previous studies [32]. While the relative crystallinity of HMT rice flours increased slightly when the rice flour mass fraction was 100% and 90%, the relative crystallinity decreased when the rice flour mass fraction was 80%. When the rice flour mass fraction was down to 70%, the relative crystallinity decreased sharply. This observation could be related to the partial gelatinization deduced from the SEM observation and explained the changes of the DSC assay, which will be discussed in Section 3.6.

Taken together, for SXG100, HMT only caused the alteration of relative crystallinity but did not change the type of starch crystalline and the relative crystallinity tended to decrease under HMT of higher intensity. In contrast, for *sbeIIb*/*Lgc1* rice with B-type semi-structure, HMT would cause the transition of the starch crystalline type.

### 3.6. Gelatinization Properties

DSC assays were conducted to investigate the changes of the gelatinization behaviors of rice flour after HMT as presented in Table 3 and Figure S4. The onset gelatinization temperature (T_o_), the peak gelatinization temperature (T_p_) and the conclusion gelatinization temperature (T_c_) of native *sbeIIb*/*Lgc1* flour were all significantly higher than those of SXG100, which is consistent with the previous study suggesting the naturally higher thermal stability of *sbeIIb*/*Lgc1* than common rice [13,14].

For HMT rice flour of *sbeIIb*/*Lgc1*, when the rice flour mass fraction was 100%, the pattern of the thermogram was similar to that of native flour (Figure S4A), while T_o_, T_p_ and ΔH were slightly lower and T_c_ was comparable with that of native flour. This observation suggested that the lower intensity HMT did not strengthen the interactions of starch molecules dramatically, but even disrupted the interactions of double helices in starch fractions with a lower thermal stability of *sbeIIb*/*Lgc1* which might be related to a slight reduction of relative crystallinity (Figure 4) and the reduction of RS content of ungelatinized HMT flour (Figure 1). When the rice flour mass fraction was 90%, the gelatinization temperatures and ΔH began to increase and the shoulder peaks of T_p_ become apparent (Figure S4A), which was also observed in the HMT samples of other cereal starches or flours in previous studies [29,33,43]. The newly formed peak indicated the formation of starch fractions with higher thermal stability including the complex between amylose and amylopectin molecules, and amylose with other components. The enthalpy of gelatinization (ΔH) signifies the molecular order of starch and has been shown to be consistent with the amount of starch double helices [29,45]. The elevated ΔH indicated the strengthened double helix structures due to the rearrangement and the enhanced hydrogen bonds of starch molecules caused by higher intensity HMT [31]. When the rice flour mass fraction was further reduced to 80%, the T_p_ corresponding to starch fractions with higher thermal stability became dominant (Figure S4A), while ΔH was lower than that of the HMT sample at 90% rice flour mass fraction, reflecting the disruption of the double helix structures in starch fractions with lower thermal stabilities. When the rice flour mass fraction was 70%, only a peak corresponding to high T_p_ was apparent (Figure S4A) and ΔH further decreased. It is consistent with the partial gelatinization as reflected by SEM observation (Figure 3), caused by the melting of ordered double helix structures of starch molecules to a greater extent [33,39].

For SXG100, when the rice flour mass fraction was 100%, the T_o_, T_p_, T_c_ and ΔH were comparable with those of native flour while slight increases were observed when the rice flour mass fraction was 90%. The patterns of thermograms were also similar to that of native flour at both rice flour mass fractions of 100% and 90% (Figure S4B). When the rice mass flour fraction was increased to 80%, the shoulder peaks of T_p_ were also observed, suggesting the formation of starch fractions with a higher thermal stability (Figure S4B). However, the ΔH decreased dramatically, indicating the occurrence of partial gelatinization [33,39]. When the rice mass flour fraction was increased to 70%, the thermogram was almost a smooth line (Figure S4B). This observation might be attributed to a greater degree of partial gelatinization [33,39].

Taken together, compared with SXG100, the native rice flour of *sbeIIb*/*Lgc1* was affected more profoundly by HMT and a higher amount of ordered starch fractions with intensified double helices structures and enhanced thermal stability formed, which might be attributed to higher AAC and lipid content of *sbeIIb*/*Lgc1* than SXG100.

### 3.7. Pasting Properties

RVA assay was conducted to analyze the changes in the pasting properties of HMT rice flour. The measured pasting parameters of the native and HMT flours were shown in Table 4. The peak viscosity (PV), the trough viscosity (TV), the final viscosity (FV), the breakdown value (BD) and the setback value (SB) of native *sbeIIb*/*Lgc1* rice flour were all significantly lower than those of SXG100, reflecting stronger resistance of *sbeIIb*/*Lgc1* starch granules to swelling than common rice due to higher content of amylose, which is congruent with numerous previous studies [13,14,26,46]. As a result of HMT, all the pasting parameters of *sbeIIb*/*Lgc1* rice flours decreased drastically, especially when the rice flour mass fraction was 80% and 70%, under which the consistency of starch might be a suspension of starch granules instead of a gel [47].

For the HMT rice flours of SXG100 with rice mass fractions of 100% to 80%, the PV and BD were also lower than those of native flour and decreased gradually as the rice flour mass fraction decreased (Table 4). Nevertheless, despite the decreasing tendency along with the decreasing of the rice flour mass fraction, the TV, the FV and the SB of SXG100 HMT rice flours with a higher rice flour mass fraction were higher than those of native rice flour (Table 4 and Figure S5), which is contrary to the results of rice with higher AAC [29,33] but is similar to those of waxy rice [6,45]. This observation might be attributed to the relatively lower AAC of SXG100 (~10%) the reason for which remains to be elucidated. At a 70% rice flour mass fraction of SXG100, all the viscosity values dropped drastically (Table 4 and Figure S5).

The reductions of the PV and BD of HMT *sbeIIb*/*Lgc1* rice flours were more severe than those of SXG100 (Table 4). The reduction in pasting values could be well attributed to the effects of HMT that strengthened the interactions of starch molecules and of starch and other components, e.g., lipids and protein, to further restrict the swelling of the starch granules and hinder the leaching of amylose during heat treatment [33,45]. The unique characteristics of starch from *sbeIIb*/*Lgc1,* including higher AAC and lipid content, might have enhanced the effects of HMT and resulted in more profound changes in PV and BD.

Combined with the results of DSC assays, HMT further enhanced the thermal stability of *sbeIIb*/*Lgc1* rice flour [29,40], suggesting that HMT *sbeIIb*/*Lgc1* rice flour might serve as a desirable ingredient of heat-processed food that is expected to have lower digestibility.

### 3.8. Protein Compositions

**Figure 5:**
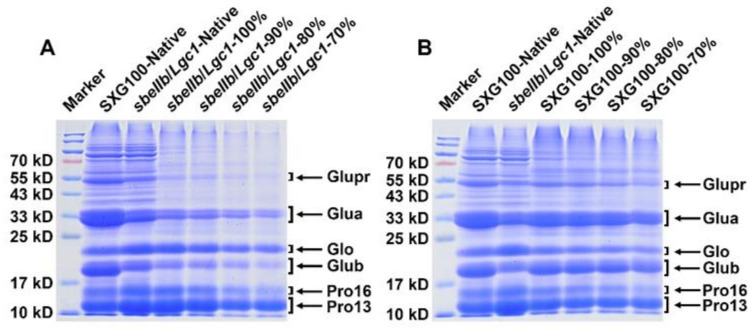
SDS-PAGE of total protein of the native and HMT flours of *sbeIIb*/*Lgc1* (**A**) and SXG100 (**B**). Marker: protein molecular weight ladder; Glupr: glutelin precursor, Glua: glutelin acidic subunit; Glub: basic subunit, Glo: Globulin, Pro16: prolamin of ~16 kD, Pro13: prolamin of ~13 kD.

Except for starch-related properties, the low glutelin content is another desired characteristic of *sbeIIb*/*Lgc1* rice. To analyze the changes of protein composition of rice flour after HMT, total protein of the native and HMT flours was extracted and separated by SDS-PAGE referring to the method described previously [38]. The relative contents of various storage protein fractions were estimated by the relative intensity of the bands with those of corresponding molecular weight. In agreement with common rice cultivars, the glutelin acidic subunit of ~37 to 39 kD and the basic subunit of ~22 to 23 kD were reflected as the two brightest bands on the SDS-PAGE gel, while the bands corresponding to the prolamin of ~13 kD and the globulin of ~26 kD were relatively weaker in SXG100 (Figure 5) [13,38]. The low-glutelin feature of *sbeIIb*/*Lgc1* native and HMT flours was verified by the significantly lower intensity of the bands corresponding to two glutelin subunits and dramatically enhanced intensity in the prolamin and globulin bands when compared to SXG100 (Figure 5). This observation revealed that the storage protein composition was not affected by HMT.

In the HMT flour samples, all the bands on the gel became weaker along with the reduction in the flour mass fraction. The fact that all the bands on the protein gel became weaker following HMT suggests a reduction in free proteins, which might be owing to the increased interactions between proteins and between starch and proteins, which reduced the solubility and digestibility of proteins during the HMT process as reported by previous studies [31,39,48,49].

## 4. Conclusions

Our study has demonstrated the feasibility of HMT as a simple approach to enhance the desirable traits of low digestibility and high RS content in rice flour by taking advantage of the intrinsic properties of *sbeIIb*/*Lgc1* rice. In this study, when the rice flour mass fraction of *sbeIIb*/*Lgc1* was 80%, which is the highest moisture content of the HMT samples, the lowest digestibility and a higher RS content were observed. Compared with common rice flour, HMT had a greater effect on the reduction of digestibility and enhancement of RS content of *sbeIIb*/*Lgc1* rice flour which might be attributed to the intrinsic features of *sbeIIb*/*Lgc1*, such as higher AAC, higher lipid content and B-type crystalline structure. HMT also caused the alterations of other physicochemical properties of *sbeIIb*/*Lgc1* rice flour including darker and browner color, alteration in the semi-crystalline structure, enhanced gelatinization temperatures and reduced pasting viscosities.

This research paved the way for potential commercial applications of this promising rice flour as a vital ingredient in functional foods. Further studies are warranted to investigate the potential improvements of the HMT *sbeIIb*/*Lgc1* rice flour on stabilizing postprandial blood sugar level when adopted as an ingredient of functional food such as meal replacement powders or yogurt, which are suitable for patients suffering diabetes.

## Figures and Tables

**Table 1:** The in vitro digestibility of native and HMT rice flours of *sbeIIb*/*Lgc1* and SXG100.

Sample	Glucose Release (mg/100 mg)		
	60 Min ^a^	120 Min	180 Min
*sbeIIb*/*Lgc1*-Native	31.43 ± 0.43 d	36.92 ± 1.02 f	40.10 ± 0.36 g
*sbeIIb*/*Lgc1*-100%	31.69 ± 0.57 d	35.79 ± 0.76 f	41.87 ± 0.33 g
*sbeIIb*/*Lgc1*-90%	22.84 ± 0.84 e	26.49 ± 0.60 g	29.64 ± 0.28 h
*sbeIIb*/*Lgc1*-80%	19.58 ± 0.80 e	23.74 ± 0.27 h	26.91 ± 0.51 h
*sbeIIb*/*Lgc1*-70%	35.07 ± 0.59 d	43.10 ± 0.87 e	48.65 ± 0.80 f
SXG100-Native	45.43 ± 2.77 b	53.47 ± 0.67 c	61.73 ±0.74 c
SXG100-100%	39.71 ± 1.38 c	49.61 ± 0.50 d	57.79 ± 1.59 d
SXG100-90%	34.60 ± 0.63 d	44.44 ± 0.60 e	53.58 ± 1.53 e
SXG100-80%	48.25 ± 0.68 b	59.35 ± 1.29 b	66.18 ± 0.99 b
SXG100-70%	69.01 ± 0.81 a	74.25 ± 0.82 a	77.98 ± 1.59 a

The analyses were repeated for three times. Data are presented as mean ± standard derivation. ANOVA and least significant difference method were used to conduct statistical analyses. Different lowercase letters represent the significant difference of values. *p* < 0.01 was considered as being significantly different. ^a^ The time represents the time point after the begin of the in vitro digestibility.

**Table 2:** The chromaticity of native and HMT rice flours of *sbeIIb*/*Lgc1* and SXG100.

Sample	L*	a*	b*	ΔE*^a^*	ΔE*^b^*	WI
*sbeIIb*/*Lgc1*-Native	96.04 ± 1.82 b	0.04 ± 0.04 a	7.18 ± 0.11 a	-	2.54	91.80
*sbeIIb*/*Lgc1*-100%	94.36 ± 1.17 b	0.84 ± 0.05 d	12.75 ± 0.16 d	5.87	3.36	86.03
*sbeIIb*/*Lgc1*-90%	92.19 ± 0.78 c	0.45 ± 0.03 c	11.20 ± 0.28 c	5.58	2.92	86.34
*sbeIIb*/*Lgc1*-80%	91.68 ± 0.63 c	1.47 ± 0.05 e	14.49 ± 0.30 f	8.63	0.80	83.23
*sbeIIb*/*Lgc1*-70%	81.18 ± 1.18 d	6.55 ± 0.17 f	25.26 ± 0.36 h	24.29	2.86	67.82
SXG100-Native	98.54 ± 0.66 a	−0.02 ± 0.02 a	6.75 ± 0.04 a	-	-	93.10
SXG100-100%	95.32 ± 1.17 b	0.29 ± 0.04 b	9.58 ± 0.10 b	4.30	-	89.33
SXG100-90%	94.74 ± 1.82 b	0.23 ± 0.04 b	9.81 ± 0.33 b	4.88	-	88.87
SXG100-80%	92.22 ± 0.85 c	1.52 ± 0.07 e	13.90 ± 0.11 e	9.67	-	84.00
SXG100-70%	79.63 ± 0.53 d	6.75 ± 0.18 g	22.86 ± 0.37 g	25.75	-	68.65

The analyses were repeated nine times. L*-value indicates darkness to lightness; a*-value indicates greenness to redness; and b*-value indicates blueness to yellowness. ΔE*^a^* indicates the total color difference between HMT rice flours of *sbeIIb*/*Lgc1* and SXG100 with corresponding native rice flour. ΔE*^b^* indicates the total color difference between *sbeIIb*/*Lgc1* rice flour and SXG100 rice flour treated at the same conditions. WI indicates the whiteness index. Data are presented as mean ± standard derivation. ANOVA and least significant difference method were used to conduct statistical analyses. Different lowercase letters represent the significant difference of values. *p* < 0.01 was considered as being significantly different.

**Table 3:** Gelatinization properties of native and HMT rice flours of *sbeIIb*/*Lgc1* and SXG100.

Sample	T_o_ (°C)	T_p_ (°C) *	T_c_ (°C)	ΔH (J/g)
*sbeIIb*/*Lgc1*-Native	72.90 ± 0.41 b	80.87 ± 0.34 a	93.96 ± 0.75 a	6.82 ± 0.07 a
*sbeIIb*/*Lgc1*-100%	70.80 ± 0.26 a	79.69 ±0.07 a	94.44 ± 0.19 a	6.49 ± 0.14 a
*sbeIIb*/*Lgc1*-90%	72.00 ± 0.39 ab	85.65 ± 0.28 b	109.21 ± 0.86 b	11.65 ± 0.90 c
*sbeIIb*/*Lgc1*-80%	81.14 ± 0.72 c	92.90 ± 0.80 c	109.85 ± 0.70 b	9.74 ± 0.43 b
*sbeIIb*/*Lgc1*-70%	88.79 ± 0.13 d	97.59 ± 0.62 d	109.81 ± 0.38 b	5.47 ± 0.11 a
SXG100-Native	59.74 ± 0.23 a	68.30 ± 0.78 a	80.81 ± 1.08 a	7.03 ± 0.07 ab
SXG100-100%	59.77 ± 0.05 a	67.63 ± 0.17 a	81.13 ± 0.39 a	6.88 ± 0.17 ab
SXG100-90%	61.05 ± 0.16 a	69.54 ± 0.46 a	82.87 ± 0.56 a	7.68 ± 0.34 a
SXG100-80%	63.49 ± 0.76 b	79.03 ± 0.17 b	89.05 ± 0.54 b	2.89 ± 0.14 d

T_o_: onset gelatinization temperature, T_p_: peak gelatinization temperature, T_c_: conclusion gelatinization temperature, ΔH: gelatinization enthalpy change. The analyses were repeated three times. Data are presented as mean ± standard derivation. ANOVA and least significant difference method were used to conduct statistical analyses. Different lowercase letters represent the significant difference of values. *p* < 0.01 was considered as being significantly different. * For T_p_, the temperatures corresponding to the main peak was recorded. For the HMT rice flour of 70% flour mass fraction of SXG100, the gelatinization temperatures were not presented since the thermogram was almost a smooth line hence the accurate gelatinization temperatures could not be recognized.

**Table 4:** Pasting properties of native and HMT rice flours of *sbeIIb*/*Lgc1* and SXG100.

Sample	PV	TV	FV	BD	SB	PT
*sbeIIb*/*Lgc1*-Native	817 ± 7 e	754 ± 13 e	1508 ± 39 e	62 ± 14 d	754 ± 27 e	7.00 ± 0.00 e
*sbeIIb*/*Lgc1*-100%	225 ± 2 g	179 ± 2 g	422 ± 6 g	47 ± 1 d	243 ± 4 f	7.00 ± 0.00 e
*sbeIIb*/*Lgc1*-90%	93 ± 1 h	71 ± 1 h	181 ± 4 h	22 ± 1 d	110 ± 3 g	7.00 ± 0.00 e
*sbeIIb*/*Lgc1*-80%	38 ± 6 h	29 ± 5 h	59 ± 9 i	9 ± 2 d	31 ± 5 h	6.98 ± 0.04 e
*sbeIIb*/*Lgc1*-70%	33 ± 2 h	25 ± 2 h	55 ± 2 i	8 ± 1 d	30 ± 3 h	6.98 ± 0.04 e
SXG100-Native	2768 ± 78 a	1344 ± 12 d	2183 ± 18 d	1424 ± 90 a	839 ± 8 d	5.62 ± 0.08 a
SXG100-100%	2648 ± 22 b	2089 ± 59 a	3869 ± 40 a	559 ± 74 b	1780 ± 28 a	5.91 ± 0.03 b
SXG100-90%	2306 ± 64 c	2001 ± 37 b	3630 ± 7 b	305 ± 35 c	1629 ± 31 b	6.13 ± 0.07 c
SXG100-80%	1982 ± 7 d	1904 ± 11 c	3248 ± 6 c	78 ± 8 d	1344 ± 8 c	6.55 ± 0.04 d
SXG100-70%	496 ± 2 f	417 ± 2 f	672 ± 5 f	80 ± 4 d	255 ± 7 f	7.00 ± 0.00 e

PV: peak viscosity, TV: trough viscosity, FV: final viscosity, BD: breakdown value, SB: setback value, PT: peak time. The analyses were repeated three times. Data are presented as mean ± standard derivation. ANOVA and least significant difference method were used to conduct statistical analyses. Different lowercase letters represent the significant difference of values. *p* < 0.01 was considered as being significantly different.

## Supplementary Materials

The following are available online at https://www.mdpi.com/article/10.3390/foods10112562/s1, Figure S1. The appearance of freshly steamed rice grains of *sbeIIb*/*Lgc1* and SXG100. Figure S2. Processed X-ray diffraction patterns of the native and HMT rice flours of *sbeIIb*/*Lgc1*. The X-axes represent the 2θ diffraction angle (°), and the Y-axes represent the relative intensity. For individual sample, the upper panel indicates the XRD pattern with subtraction of the background (upper pattern), and the diffraction peaks (lower pattern), and the lower panel indicates the resolution of multiple diffraction peaks. Figure S3. Processed X-ray diffraction patterns of the native and HMT rice flours of SXG100. The X-axes represent the 2θ diffraction angle (°), and the Y-axes represent the relative intensity. For individual sample, the upper panel indicates the XRD pattern with subtraction of the background (upper pattern), and the diffraction peaks (lower pattern), and the lower panel indicates the resolution of multiple diffraction peaks. Figure S4. DSC thermograms of the native and HMT rice flours of *sbeIIb*/*Lgc1* (A) and SXG100 (B). The analysis of each sample was carried out with three replications and one representative DSC thermogram was presented. Figure S5. The pasting curves of native and HMT rice flours of *sbeIIb*/*Lgc1* and SXG100. The analysis of each sample was carried out with three replications and one representative pasting RVA curve was presented. 1~5 represent *sbeIIb*/*Lgc1*-Native, 100%, 90%, 80% and 70% respectively; 6~10 represent SXG100-Native, 100%, 90%, 80% and 70% respectively. The green line presents the temperature change along with the heat time.
