# Supplementary material for: Heat-Moisture Treatment Further Reduces In Vitro Digestibility and Enhances Resistant Starch Content of a High-Resistant Starch and Low-Glutelin Rice
Source: Foods. 2021 Oct 24;10(11):2562. doi: 10.3390/foods10112562 (PMC8622339; doi:10.3390/foods10112562)
Supplement: Supplementary file 1 [file foods-10-02562-s001.zip › foods-1407521-SI.pdf]

# Heat-Moisture Treatment Further Reduces In Vitro Digestibility and Enhances Resistant Starch Content of a High-Resistant Starch and Low-Glutelin Rice

**Zhiyuan Li [1,2,#], Dongshu Guo [2,#], Xiao Li [2], Zhaocheng Tang [2], Xitie Ling [2], Tiantian Zhou [2] and Baolong Zhang [1,2,*]**

[1] School of Food and Biological Engineering, Jiangsu University, Zhenjiang 212013, China.

[2] Excellence and innovation center, Jiangsu Academy of Agricultural Science, Nanjing 210014, China.

# These two authors contribute equally to this work.

* Corresponding authors:

Prof. Dr. Baolong Zhang. Tel.: +86-25-84390292. E-mail: zhbl2248@hotmail.com

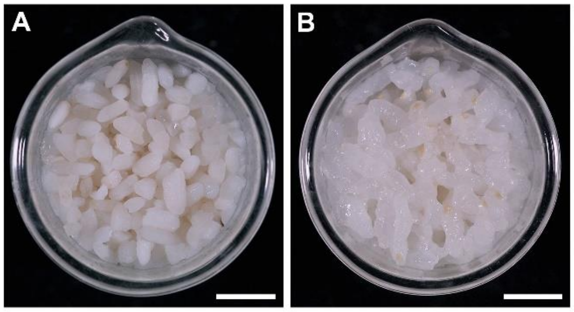


**Figure S1. The appearance of freshly steamed rice grains of *sbeIIb/Lgc1* and SXG100.** (A) *sbeIIb/Lgc1*, (B) SXG100. Bars = 1 cm.

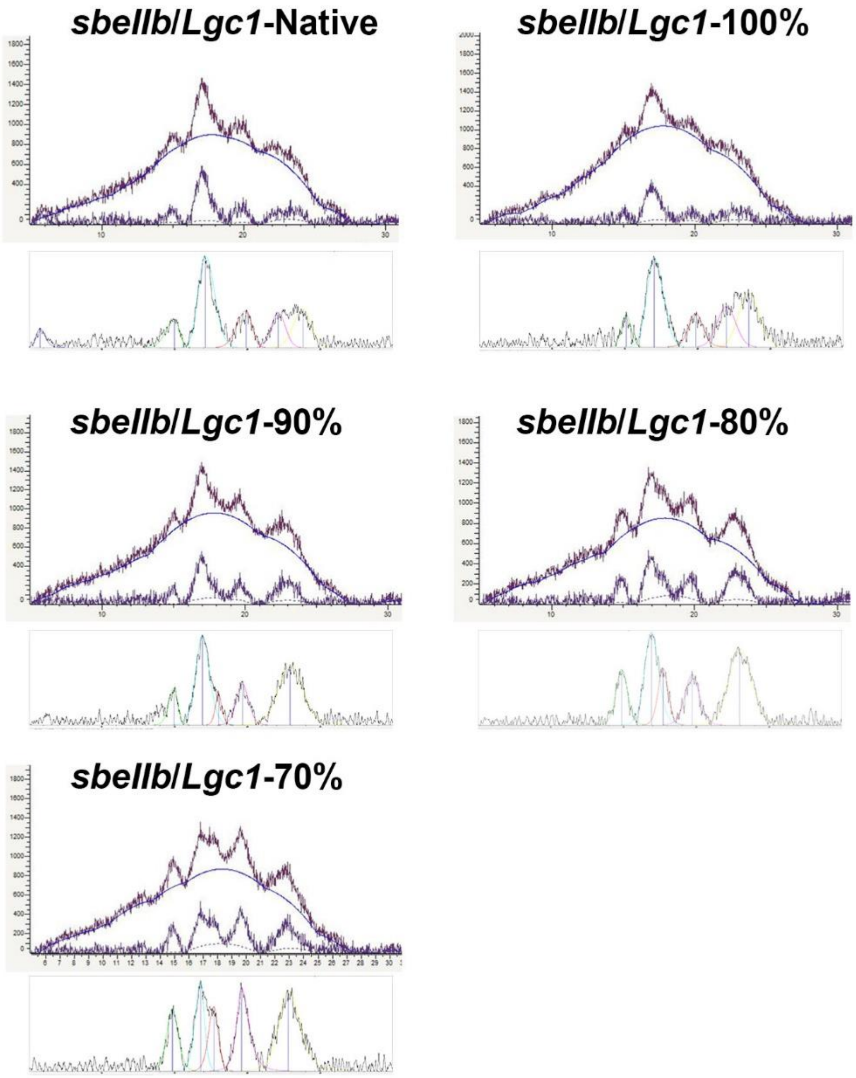


**Figure S2.** Processed X-ray diffraction patterns of the native and HMT rice flours of *sbeIIb*/*Lgc1*. The X-axes represent the 2θ diffraction angle (°), and the Y-axes represent the relative intensity. For individual sample, the upper panel indicates the XRD pattern with subtraction of the background (upper pattern), and the diffraction peaks (lower pattern), and the lower panel indicates the resolution of multiple diffraction peaks.

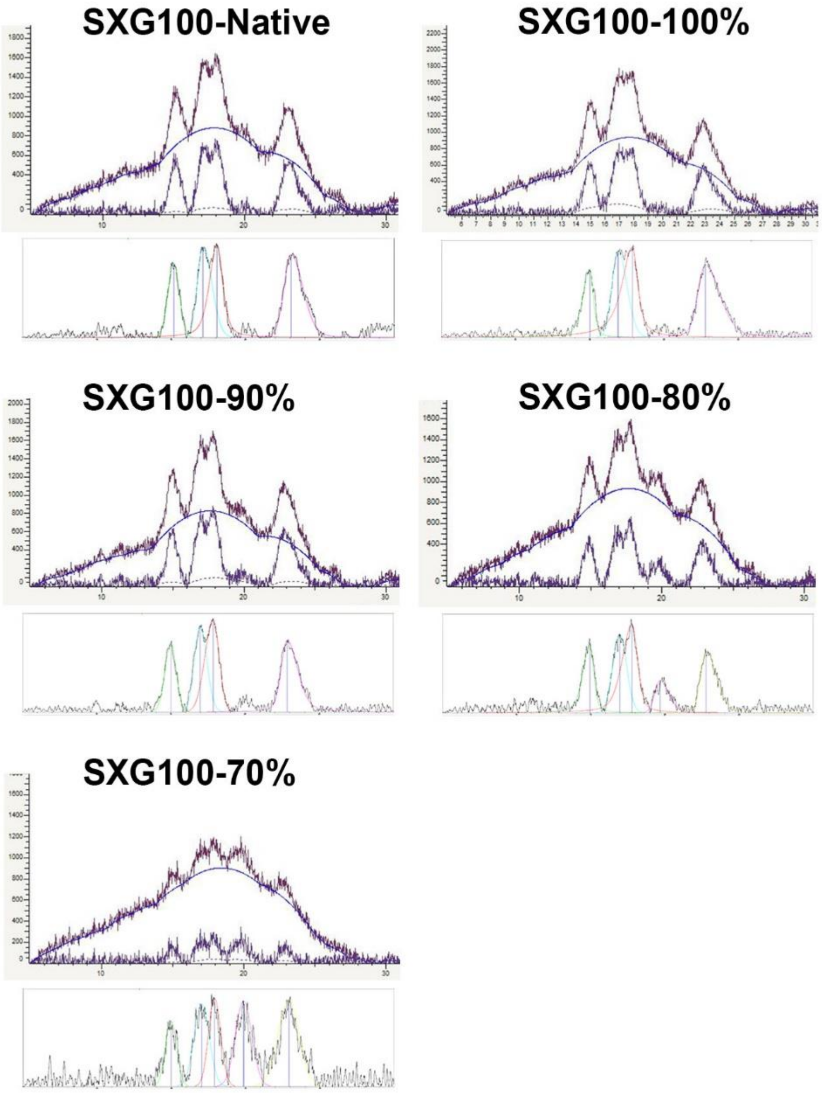


**Figure S3.** Processed X-ray diffraction patterns of the native and HMT rice flours of SXG100. The X-axes represent the 2θ diffraction angle (°), and the Y-axes represent the relative intensity. For individual sample, the upper panel indicates the XRD pattern with subtraction of the background (upper pattern), and the diffraction peaks (lower pattern), and the lower panel indicates the resolution of multiple diffraction peaks.

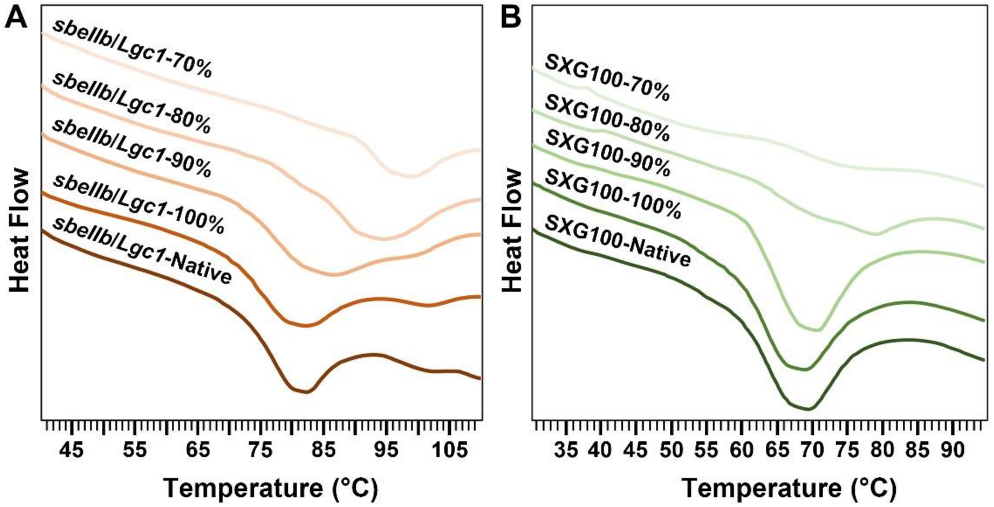


**Figure S4.** DSC thermograms of the native and HMT rice flours of *sbeIIb/Lgc1* (A) and SXG100 (B). The analysis of each sample was carried out with three replications and one representative DSC thermogram was presented.

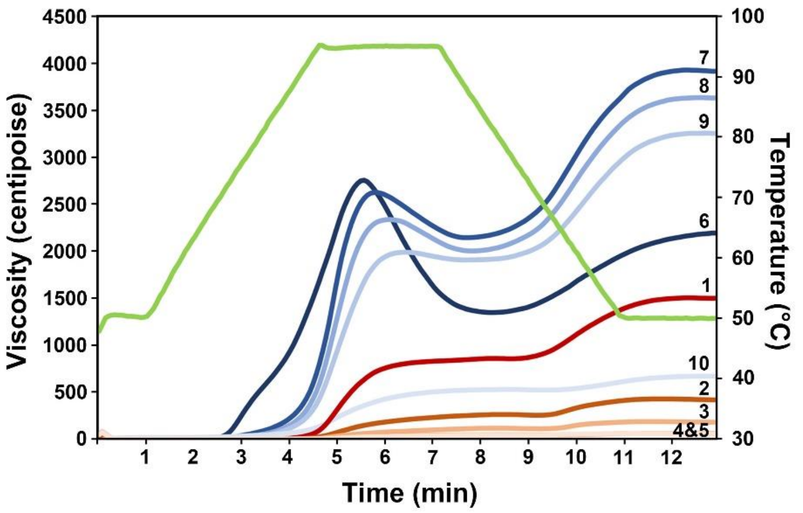


**Figure S5.** The pasting curves of native and HMT rice flours of *sbeIIb*/*Lgc1* and SXG100. The analysis of each sample was carried out with three replications and one representative pasting RVA curve was presented. 1~5 represent *sbeIIb*/*Lgc1*-Native, 100%, 90%, 80% and 70% respectively; 6~10 represent SXG100-Native, 100%, 90%, 80% and 70% respectively. The green line presents the temperature change along with the heat time.
